# Gene Expression Essential for Myostatin Signaling and Skeletal Muscle Development Is Associated With Divergent Feed Efficiency in Pedigree Male Broilers

**DOI:** 10.3389/fphys.2019.00126

**Published:** 2019-02-26

**Authors:** Kentu Lassiter, Byungwhi Caleb Kong, Alissa Piekarski-Welsher, Sami Dridi, Walter Gay Bottje

**Affiliations:** ^1^Department of Poultry Science, Center of Excellence for Poultry Science, University of Arkansas, Fayetteville, AR, United States; ^2^Adisseo USA, Alpharetta, GA, United States

**Keywords:** feed efficiency, muscle development, myostatin signaling, pedigree male broilers, gene expression

## Abstract

**Background:** Feed efficiency (FE, gain to feed) is an important genetic trait as 70% of the cost of raising animals is due to feed costs. The objective of this study was to determine mRNA expression of genes involved in muscle development and hypertrophy, and the insulin receptor-signaling pathway in breast muscle associated with the phenotypic expression of FE.

**Methods:** Breast muscle samples were obtained from Pedigree Male (PedM) broilers (8 to 10 week old) that had been individually phenotyped for FE between 6 and 7 week of age. The high FE group gained more weight but consumed the same amount of feed compared to the low FE group. Total RNA was extracted from breast muscle (*n* = 6 per group) and mRNA expression of target genes was determined by real-time quantitative PCR.

**Results:** Targeted gene expression analysis in breast muscle of the high FE phenotype revealed that muscle development may be fostered in the high FE PedM phenotype by down-regulation several components of the myostatin signaling pathway genes combined with upregulation of genes that enhance muscle formation and growth. There was also evidence of genetic architecture that would foster muscle protein synthesis in the high FE phenotype. A clear indication of differences in insulin signaling between high and low FE phenotypes was not apparent in this study.

**Conclusion:** These findings indicate that a gene expression architecture is present in breast muscle of PedM broilers exhibiting high FE that would support enhanced muscle development-differentiation as well as protein synthesis compared to PedM broilers exhibiting low FE.

## Introduction

Feed efficiency (FE) is one of the most important genetic traits in animal production since feed costs roughly 70% of the total cost required to bring an animal to market weight (Willems et al., 2013). In a Pedigree Male (PedM) broiler FE model, animals with Low FE exhibited higher mitochondrial reactive oxygen species (ROS) production and higher oxidative stress compared to those with high FE (Bottje and Carstens, 2009; Bottje and Kong, 2013). Although ROS causes oxidation at high levels, low levels of mitochondrial ROS are important in signal transduction and control various physiological processes affecting cell growth and proliferation (Finkel, 2011; Bleier et al., 2015). Therefore, we hypothesized that inherent gene expression in the low FE phenotype was modulated by mitochondrial ROS production (Bottje et al., 2002; Kong et al., 2011).

To develop a comprehensive understanding of the cellular basis of FE, global gene and protein expression studies have been conducted on breast muscle obtained from PedM broilers exhibiting high and low FE phenotypes (Kong et al., 2011, 2016; Bottje et al., 2017a). These studies have provided insight into fundamental mechanisms of FE in muscle but have also provided certain paradoxical findings. For example, despite clear enrichment of cytoskeletal or muscle fiber gene expression occurring in the low FE phenotype (Kong et al., 2011; Bottje et al., 2017a), myostatin (MSTN), that inhibits muscle development (McPherron and Lee, 1997; McPherron et al., 1997; Kollias and McDermott, 2008), was up-regulated in breast muscle of low FE PedM broilers (Bottje et al., 2014). The expression of adenosine monophosphate-activated protein kinase 1 (AMPK), a sensor of energetic status in the cell, was elevated in a cDNA microarray in muscle of the high FE PedM phenotype (Bottje et al., 2012). When phosphorylated, AMPK activates energy production pathways (e.g., glycolysis, lipolysis, oxidative phosphorylation) and inhibits energy-consuming pathways (e.g., lipogenesis, protein synthesis) (Hardie, 2007; Jeon, 2016). Thus, increased AMPK activity in the high FE phenotype could inhibit protein synthesis and would appear to run counter to the fact that these animals gained more body weight that would require increased protein synthesis to support tissue accretion in comparison to the low FE PedM phenotype (Bottje et al., 2002; Kong et al., 2011). Therefore, the objective of this study was to conduct targeted analysis of genes involved in muscle development, protein synthesis, and energy metabolism; particularly with genes associated with the myostatin and insulin signaling pathways in breast muscle obtained from PedM broilers exhibiting a high or low FE phenotype.

## Materials and Methods

### Ethics Statement

This study was conducted in accordance with the recommendations in the guide for the care and use of laboratory animals of the National Institutes of Health. All procedures for animal care were reviewed and approved by the University of Arkansas Institutional Animal Care and Use Committee (IACUC: Protocol #14012).

### Tissues-Animals

Breast muscle samples analyzed in the present study were obtained from PedM broilers during an earlier study (Bottje et al., 2009) that had been individually phenotyped for FE as previously described (Bottje et al., 2002). Briefly, FE (amount of body weight gain/amount of feed consumed) was determined between 6 and 7 week of age on a group of 100 PedM broilers housed in individual cages (51 cm × 51 cm × 61 cm) at thermoneutral temperatures. Birds were provided access to feed (corn-soybean based diet; 20.5% protein and 3,280 kcal/kg) and water *ad libitum* during and after the week of FE phenotyping. From the initial group of 100, birds with the highest and lowest FE were selected. Feed efficiencies for the low- and high-FE groups (*n* = 6 per group) were 0.46 + 0.01 and 0.65 + 0.01, respectively. In the high FE group, greater efficiency was attained by greater weight gain without a difference in feed intake during the week of phenotyping. The difference in mean FE (0.19) between the groups is identical to that reported in our initial investigation (Bottje et al., 2002). The birds were then transported from the commercial breeding farm where FE phenotyping took place to the University of Arkansas and placed in cages under similar conditions. Following a 4 day acclimation period, birds were humanely euthanized, breast muscle tissue (*Pectoralis major*) was quickly excised and flash frozen in liquid nitrogen, and stored at −80°C. Tissue samples were obtained from these birds between 8 and 10 weeks of age associated with mitochondrial function (proton leak kinetic studies) (Bottje et al., 2009). We have verified tissue sample quality in previous transcriptomic and proteomic studies (Kong et al., 2011, 2016; Bottje et al., 2017a).

### RNA Isolation and Quantitative Real-Time PCR

The integrity of mRNA samples were verified by agarose gel electrophoresis by the presence of three clear and discrete bands representing 5S, 18S, and 28S ribosomal subunits. This assessment was made in the present study as well as in previous transcriptomic studies (Kong et al., 2011; Bottje et al., 2017a). Total RNA was extracted by TRIzol reagent (Life Technologies, Thermo Fisher Scientific, Carlsbad, CA, United States) according to the manufacturer’s recommendations, DNase-treated, and reverse-transcribed (Quanta Biosciences, Gaithersburg, MD, United States). The RNA isolated in the present study were from tissue maintained at −80°C and not previously thawed. The concentration, purity, and quality of RNA in the present study were determined for each sample using a Take 3 microvolume plate and a Synergy HT multimode microplate reader (BioTek, Winooski, VT, United States). The reverse-transcription (RT) products (cDNAs) were amplified by real-time quantitative PCR (7500 real-time PCR system, Applied Biosystems, Thermo Fisher Scientific, Foster City, CA, United States) with Power SYBR Green Master Mix (Applied Biosystems). Oligonucleotide primers used in this study, summarized in Table 1, include: chicken activin receptor types IIA and IIB (ActRIIA and ActRIIB), activin-like kinases 4 and 5 (ALK4 and ALK5), AMP-activated protein kinase alpha subunits 1 and 2 (AMPKα1 and AMPKα2), caveolin-3 (CAV-3), creatine kinase muscle isoform (CKM), follistatin (FSTN), glucose transporter 8 (GLUT-8), heat shock 70 kDa protein (HSP70), insulin degrading enzyme (IDE), insulin-like growth factor 1 (IGF-1), insulin-like growth factor-binding protein 3 (IGFBP-3), insulin receptor (IR), insulin receptor substrate 1 (IRS-1), mitogen-activated protein kinases 1, 2, 4, and 6 (MAP2K1, MAP2K2, MAP2K4, and MAP2K6), mitogen-activated protein kinase 7 (MAP3K7), myostatin (MSTN), mechanistic target of rapamycin (mTOR), myogenin (MYOG), myozenin 2 (MYOZ-2), neutrophil cytosolic factor 2 (NCF2), 70 kDa ribosomal protein S6 kinase (P70S6K), cAMP-dependent protein kinase type I-alpha regulatory subunit (PRKAR1A), regulatory-associated protein of mTOR (RAPTOR), SHC-transforming protein 1 (SHC-1), mothers against decapentaplegic homologs 2 and 3 (SMAD2 and SMAD3), and the housekeeping gene ribosomal 18S.

**Table 1:** Oligonucleotide for quantitative reverse transcription polymerase chain reaction (qRT-PCR) primers.

Gene symbol	Gene name	Accession number	Primer sequence (5′ → 3′)	Orientation	Product size (bp)
ActRIIA	Activin	NM_205367.1	GCCATCTCACACAGGGACAT	Forward	146
	receptor IIA		TACCTTCGTGTGCCAACCTG	Reverse	
ActRIIB	Activin	NM_204317.1	CGTGACCATCGAAGAGTGCT	Forward	130
	receptor IIB		CACGATGGAGACAAGGCAGT	Reverse	
ALK4	Activin-like	XM_001231300.3	CCGCTACACGGTGACCATAG	Forward	107
	kinase 4		TCCCAGGCTTTCCCTGAGTA	Reverse	
ALK5	Actvin-like	NM_204246.1	GGCAGAGCTGTGAGGCATTA	Forward	73
	kinase 5		CTAGCAGCTCCGTTGGCATA	Reverse	
AMPKα1	AMP activated	NM_001039603.1	CCACCCCTGTACCGGAAATA	Forward	68
	kinase a 1		GGAAGCGAGTGCCAGAGTTC	Reverse	
AMPKα2	AMP activated	NM_001039605.1	TGTAAGCATGGACGTGTTGAAGA	Forward	62
	kinase a 2		GCGGAGAGAATCTGCTGGAA	Reverse	
CAV-3	Caveolin-3	NM_204370.2	CGTTGTAAAGGTGGATTTCGAGG	Forward	110
			ACCAGTACTTGCTGACGGTG	Reverse	
CK(m)	Creatine kinase	NM_205507.1	TGGGTTACATCCTGACGTGC	Forward	101
	(muscle isoform)		CTCCTCGAATTTGGGGTGCT	Reverse	
FSTN	Follistatin	NM_205200.1	CCCGGGCATGCTCGTA	Forward	60
			TGCGCTGTGTGATCTTCCAT	Reverse	
GLUT-8	Glucose	NM_204375.1	GGCATCGTGGTTTGGGTCTA	Forward	73
	transporter-8		ATCCACAAGGTAGCCTCCCA	Reverse	
HSP-70	Heat shock	JO2579	GGGAGAGGGTTGGGCTAGAG	Forward	55
	protein 70		TTGCCTCCTGCCCAATCA	Reverse	
IDE	Insulin degrading	XM_421686.5	GCCCATTTGCTTACGTGGAT	Forward	77
	enzyme		GTTGAGGGAGTCTTTGAGTAGTTCAA	Reverse	
IGF-1	Insulin-like	NM_001004384.2	GCTGCCGGCCCAGAA	Forward	56
	growth factor 1		ACGAACTGAAGAGCATCAACCA	Reverse	
IGFBP-3	IGF-1	NM_001101034.1	ATCAGGCCATCCCAAGCTT	Forward	59
	binding protein		GATGTGCTGTGGAGGCAAATT	Reverse	
IRS-1	Insulin receptor	NM_005544.2	GCGCAAGGTGGGCTACCT	Forward	64
	substrate 1		CGCGCGCAGTACGAAGA	Reverse	
MAP2K6	Mitogen activated	XM_003642348.2	TGTCTCAGTCGAGAGGCAAA	Forward	105
	kinase 6		TGGAGTCTAGATCCCTGGGT	Reverse	
MAP3K7	Mitogen activated	XM_004940375.1	CCTGATGATGCAGGTAAGACCA	Forward	107
	protein kinase 7		TCTTTGGAGTTCGGGCATGG	Reverse	
MSTN	Myostatin	NM_001001461.1	ATGCAGATCGCGGTTGATC	Forward	59
			GCGTTCTCTGTGGGCTGACT	Reverse	
mTOR	Mechanistic target	XM_417614.5	CATGTCAGGCACTGTGTCTATTCTC	Forward	77
	of rapamycin		CTTTCGCCCTTGTTTCTTCACT	Reverse	
MYOG	Myogenin	NM_204184.1	GGAGAAGCGGAGGCTGAAG	Forward	62
			GCAGAGTGCTGCGTTTCAGA	Reverse	
MYOZ2	Myozenin	NM_001277827.1	CAACACTCAGCAACAGAGGC	Forward	120
			GTATGGGCTCTCCACGATTTCT	Reverse	
NCF2	Neutrophil	XM_004943279.1	TCTTTGCTTGCGAGGTGGT	Forward	111
	cytosolic factor 2		TTTCTGGTGTCTTGGGCCTG	Reverse	
P70S6K	70 kDa ribosomal	NM_001109771.2	GTCAGACATCACTTGGGTAGAGAAAG	Forward	60
	Protein S6 kinase		ACGCCCTCGCCCTTGT	Reverse	
PRKAR1A	cAMP dependent	NM_001007845.1	GTGGGAGCGCCTTACTGTAG	Forward	119
	kinase Ia		CAGCTGTGCCCTCCAAGATA	Reverse	
RAPTOR	Regulatory protein	XM_004946275.1	GGCTACGAGCTCTGGATCTG	Forward	70
	of mTOR		TGACATGACAAGCTAACTGCC	Reverse	
SHC-1	SHC-transforming	NM_001293280.1	CTGCTCAAGCAGGAAGAGAGAAA	Forward	110
	protein 1		GCGTGTCTTGTCCACGTTCT	Reverse	
SMAD2	Mothers against	NM_204561.1	TGAGTATAGGCGGCAGACCG	Forward	107
	decapentaplegic homolog 2		AAGGGGAGCCCATCTGAGTC	Reverse	
SMAD3	Mothers against	NM_204475.1	CCCACCGTTGGACGATTACA	Forward	99
	decaplegic homolog 3		GGAGGAGGTGTCTCTGGGAT	Reverse	
18S		AF173612	TCCCCTCCCGTTACTTGGAT	Forward	60
			GCGCTCGTCGGCATGTA	Reverse	

### Statistical Analysis

Comparison of mean expression values for qRT-PCR between the high and low FE groups were made using Student’s *t*-test. Differences were considered statistically significant at *P* < 0.05 with qualified significance provided for *P*-values greater than 0.05 but less than 0.10. Binomial distribution analysis was used as previously described to assess differences in the number of genes associated with eukaryotic initiation and translation factors involved in protein synthesis of unreported data (see Table 2) contained in a transcriptomic dataset (Bottje et al., 2017a). Briefly, the numbers of molecules in which average values were numerically higher (H) or lower (L) in breast muscle of the high FE compared to the low FE PedM phenotype were determined and used in the exact binomial distribution analysis test offered in the 2010 version of Microsoft Excel^TM^. There was no cutoff based on significant or fold difference in expression for a given transcript involved in the bionomial distribution analysis that was conducted.

**Table 2:** Expression of eukaryotic translation elongation and initiation factors list obtained from an RNAseq dataset of breast muscle tissue (from Bottje et al., 2017a) showing log_2_ high feed efficiency – log_2_ low feed efficiency (M), the gene symbol and the gene name.

*M*	Symbol	Entrez gene name
−0.67	EEF1A1	Eukaryotic translation elongation factor 1 alpha 1
−0.18	EIF6	Eukaryotic translation initiation factor 6
−0.17	EIF4A2	Eukaryotic translation initiation factor 4A2
−0.15	EIF2AK2	Eukaryotic translation initiation factor 2 alpha kinase 2
−0.11	EIF2AK3	Eukaryotic translation initiation factor 2 alpha kinase 3
−0.10	EIF3H	Eukaryotic translation initiation factor 3 subunit H
−0.07	EIF3E	Eukaryotic translation initiation factor 3 subunit E
−0.07	EIF3M	Eukaryotic translation initiation factor 3 subunit M
−0.05	EIF2AK1	Eukaryotic translation initiation factor 2 alpha kinase 1
−0.05	EIF4H	Eukaryotic translation initiation factor 4H
−0.04	EIF3L	Eukaryotic translation initiation factor 3 subunit L
−0.03	EIF4A3	Eukaryotic translation initiation factor 4A3
−0.01	EIF4ENIF1	Eukaryotic translation initiation factor 4E nuclear import factor 1
0.00	EIF2S3	Eukaryotic translation initiation factor 2 subunit gamma
0.00	EIF3A	Eukaryotic translation initiation factor 3 subunit A
0.02	EEF1AKMT1	Eukaryotic translation elongation factor 1 alpha lysine methyltransferase 1
0.04	EEF2	Eukaryotic translation elongation factor 2
0.05	EEF1A1	Eukaryotic translation elongation factor 1 alpha 1
0.05	EIF2B5	Eukaryotic translation initiation factor 2B subunit epsilon
0.05	EIF4E	Eukaryotic translation initiation factor4E
0.06	EIF3I	Eukaryotic translation initiation factor 3 subunit I
0.06	EIF4G2	Eukaryotic translation initiation factor 4 gamma 2
0.06	EIF5A2	Eukaryotic translation initiation factor 5A2
0.07	EIF2B1	Eukaryotic translation initiation factor 2B subunit alpha
0.08	EIF4E3	Eukaryotic translation initiation factor 4E family member 3
0.10	EIF1B	Eukaryotic translation initiation factor 1B
0.10	EIF3B	Eukaryotic translation initiation factor 3 subunit B
0.11	EIF2D	Eukaryotic translation initiation factor 2D
0.11	EIF3F	Eukaryotic translation initiation factor 3 subunit F
0.12	CTIF	Cap binding complex dependent translation initiation factor
0.12	EIF4E2	Eukaryotic translation initiation factor 4E family member 2
0.14	EIF2AK4	Eukaryotic translation initiation factor 2 alpha kinase 4
0.14	HBS1L	HBS1 like translational GTPase
0.17	EIF4G3	Eukaryotic translation initiation factor 4 gamma 3
0.17	TPT1	Tumor protein, translationally-controlled 1
0.18	EIF1AY	Eukaryotic translation initiation factor 1A, Y-linked
0.19	EIF1	Eukaryotic translation initiation factor 1
0.19	EIF3D	Eukaryotic translation initiation factor 3 subunit D
0.21	EIF2B2	Eukaryotic translation initiation factor 2B subunit beta
0.21	EIF4G1	Eukaryotic translation initiation factor 4 gamma 1
0.22	EEF1D	Eukaryotic translation elongation factor 1 delta
0.22	EIF2B3	Eukaryotic translation initiation factor 2B subunit gamma
0.23	MTO1	Mitochondrial tRNA translation optimization 1
0.24	EIF2B1	Eukaryotic translation initiation factor 2B subunit alpha
0.24	EIF5	Eukaryotic translation initiation factor 5
0.25	EIF2B4	Eukaryotic translation initiation factor 2B subunit delta
0.26	TMA16	Translation machinery associated 16 homolog
0.28	EIF2A	Eukaryotic translation initiation factor 2A
0.28	EIF2S1	Eukaryotic translation initiation factor 2 subunit alpha
0.28	EIF5B	Eukaryotic translation initiation factor 5B
0.29	EEF1B2	Eukaryotic translation elongation factor 1 beta 2
0.32	EIF3J	Eukaryotic translation initiation factor 3 subunit J
0.33	MTIF2	Mitochondrial translational initiation factor 2
0.41	EIF4EBP1	Eukaryotic translation initiation factor 4E binding protein 1
0.43	MTIF3	Mitochondrial translational initiation factor 3
0.45	MTRF1	Mitochondrial translational release factor 1
0.61	MSS51	MSS51 mitochondrial translational activator
0.66	MTRF1L	Mitochondrial translational release factor 1 like

*A negative or positive M-value indicates expression was numerically lower or higher in the high feed efficiency phenotype compared to the low feed efficiency phenotype. Of the 56 sequences that were detected, 13 were lower (negative M-value) in the high feed efficiency phenotype and 43 were higher (positive M-value) in the high FE compared to the low FE phenotype. The skew in the high FE phenotype was significant (binomial P-value = 0.0002).*

## Results and Discussion

The major goal of this study was to conduct targeted gene expression analysis using qRT-PCR to extend the understanding of fundamental mechanisms of FE in breast muscle of PedM broilers exhibiting high and low FE phenotypes. Following a brief overview of the tissues analyzed in this study, the discussion below will be directed to results obtained in the following areas: (a) muscle development and myostatin signaling, (b) energy-nutrient sensing, (c) protein synthesis, and (d) insulin signaling.

### Tissues

The tissues analyzed in the present study have been stored at −80°F since they were collected during experiments examining proton leak kinetics in PedM broilers exhibiting high and low FE phenotypes (Bottje et al., 2009) and have been intensively examined in transcriptomic studies (Kong et al., 2011; Bottje et al., 2012, 2017b) and in proteomic studies (Kong et al., 2016). These analyses, from the same set of animals, have afforded an in-depth and unique examination of molecular expression associated with the phenotypic expression of FE.

Long-term storage of frozen tissue can result in deterioration in quality of RNA and proteins. However, as indicated above, the quality of RNA that was isolated in the present study remained high as it did in previous transcriptomic studies. Protein quality was sufficient to conduct shotgun proteomics on this set of tissues (Kong et al., 2016) but an inability to detect phosphorylated proteins (unpublished observations), and potentially other post-translational modifications, is unfortunate and necessitates the reliance of software such as Ingenuity Pathway Analysis (IPA) (Qiagen, Valencia, CA, United States) to make predictions of activation or inhibition of upstream molecules based on the expression of downstream molecules in the global gene and protein expression datasets (Kong et al., 2011, 2016; Bottje et al., 2012, 2017c).

### Muscle Development and Myostatin Signaling

**FIGURE 1:**
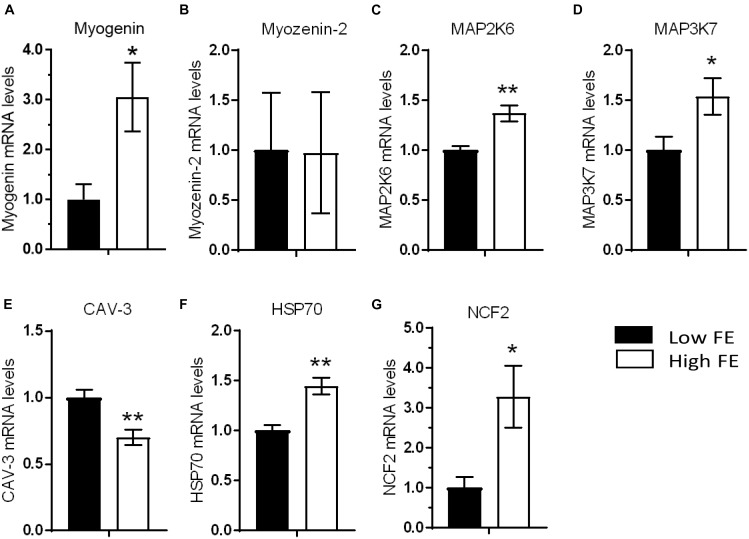
Differentially expressed genes that are involved in muscle development and differentiation in breast muscle from Pedigree Male (PedM) broilers exhibiting high or low feed efficiency (FE) phenotype. Relative expression of mRNA is shown for myogenin **(A)**, myozenin-2 **(B)**, mitogen activated protein kinase 6 (MAP2K6) **(C)**, MAP kinase 7 (MAP3K7) **(D)**, caveolin 3 (CAV-3) **(E)**, heat shock protein 70 (HSP70) **(F)**, and neutrophil cytosolic factor 2 (NCF2) **(G)**. Bars represent the mean + SE (*n* = 5). Mean values were different at ^∗^*P* ≤ 0.05 or ^∗∗^*P* ≤ 0.01.

The mRNA expression of MYOG, MAP2K6, MAP3K7, HSP70, and NCF2, genes that promote muscle development and differentiation, were higher in high FE compared to low FE PedM broilers (Figure 1). While MYOG is a key regulatory transcription factor involved in muscle development during myogenesis (Weintraub et al., 1991; Ohkawa et al., 2007; Faralli and Dilworth, 2012), there are also reports of a role of MYOG playing important roles in muscle biology after myogenesis has been completed. For example, a positive relationship between increased breast muscle weight and increased mRNA levels of MYOG in 38 d old broilers has been reported (Xiao et al., 2017). Myogenin directs satellite cell activity during muscle regeneration (Zammit, 2017) and MYOG levels increase in human skeletal muscle following denervation (Chen et al., 2011). Myogenin was also instrumental in sustaining mitochondrial activity in exhaustive exercise in mice (Flynn et al., 2011). In this regard, increased MYOG expression might be a contributing factor to enhanced mitochondrial function in the high FE PedM phenotype compared to the low FE phenotype (Bottje et al., 2002). Thus, although MYOG is primarily considered as a promoter of myogenesis, MYOG may also be important in energetics and maintenance of fully developed muscle. Increased MYOG mRNA expression could therefore be hypothesized to play a role in the phenotypic expression of FE. Myozenin (Myoz-2) encodes a protein associated with actin and myosin found on the z-line in skeletal muscle that is part of the contractile apparatus (Takada et al., 2001). Although Myoz-2 expression was down-regulated in breast muscle of the high FE PedM broiler phenotype in a recent transcriptomic study conducted on the same set of tissues (Bottje et al., 2017a), Myoz-2 was not differentially expressed in the present study (Figure 1B).

Expression of two mitogen activated kinase enzymes, MAP2K6 and MAP3K7 involved in muscle development and response to environmental stress (see review by Davis, 2000), were elevated in the high FE phenotype (Figure 1C,D). Specifically, MAP2K6 activates p38 MAP kinase and plays an important part in its signal transduction pathway (Raingeaud et al., 1996) whereas MAP3K7 is involved in activation of nuclear factor kappa β (NFκB) in addition to other MAP kinases (Ninomiya-Tsuji et al., 1999). While there were no differences in mRNA expression of MAP2K1, MAP2K2, and MAP2K4, MAP4K4 was predicted to be inhibited in the low FE phenotype in a proteomic study (Kong et al., 2016).

The mRNA expression of CAV-3 was downregulated in the high FE phenotype (Figure 1E). CAV-3 is a member of the caveolin family, and serves as the muscle-specific isoform of the caveolin protein (Biederer et al., 1998). Mutations and expression differences of CAV-3 can result in certain muscle myopathies (Woodman et al., 2004). Caveolae are sub-cellular structures that function in cell signaling by aiding internalization of hormonal signals after the hormone binds to the target receptor on the cell surface. Zhu et al. (2006) indicated that CAV-3 expression was upregulated during muscle hyperplasia when compared to expression levels during hypertrophy in pigs and hypothesized that CAV-3 might be used as a genetic marker for meat production in swine. Altered CAV-3 expression may be detrimental to muscle development as both increased or decreased CAV-3 expression made mouse muscle cells more susceptible to oxidative stress and decreased survival through PI(3)K/Akt signaling (Smythe and Rando, 2006). Thus, the up regulation of CAV-3 in the low FE phenotype compared to the low FE phenotype would potentially enhance muscle development but might have contributed to higher oxidative stress observed in the low FE PedM broiler phenotype (Bottje and Carstens, 2009). Since the expression of CAV-1 protein is involved in insulin signaling (see below) and was elevated in the high FE breast muscle (Kong et al., 2016), it is not clear what role the CAV1 and CAV3 genes are contributing to the PedM broiler FE model in the present study.

Heat shock protein 70 kDa (HSP70) mRNA expression was elevated in the high FE breast muscle (Figure 1F). This upregulation could have a positive effect in muscle as HSP70 maintains muscle fiber integrity and enhances muscle regeneration and recovery from damage (Senf, 2013). In mitochondria, HSP70 is also responsible for correct folding and assembly of nuclear-encoded proteins targeted for import into the mitochondria where they assemble with mitochondrial DNA encoded proteins as components of the mitochondrial electron transport chain (Truscott et al., 2003) and an important chaperone for mitochondrial DNA-encoded proteins (Hermann et al., 1994). Previously, we reported that HSP90 gene expression was elevated in muscle of the low FE phenotype and hypothesized this elevation was a response to higher oxidative stress in these animals (Bottje et al., 2012), whereas HSPB2 (heat shock p27 kDa protein 2) protein expression was elevated in the high FE breast muscle (Kong et al., 2016). Thus, there appears to be distinct differences in expression of different members of the family of heat shock proteins in the high and low FE phenotypes.

Neutrophil cytosolic factor 2 (NCF2) encodes a subunit of NADPH/NADH oxidase and is a critical component of NADPH oxidase 2 (NOX2) (Ferreira and Laitano, 2016). NCF2 was elevated in high FE breast muscle in the present study (Figure 1G) which concurs with findings in commercial broilers (Zhou et al., 2015). NOX2 generates superoxide in the sarcoplasmic reticulum and is a major source of oxidative stress in muscle (Dikalov, 2011; Ferreira and Laitano, 2016). NADPH oxidase also functions in the generation of superoxide in neutrophils during phagocytosis. NOX2 is a downstream target of nuclear factor erythroid 2-like 2 (NFE2L2) that coordinates antioxidant response to oxidative stress by activating expression of genes that contain an antioxidant response element in their promoter regions (e.g., Kobayashi et al., 2004, 2006). From downstream target expression analysis, NFE2L2 was predicted to be activated in animals with high FE (Zhou et al., 2015; Kong et al., 2016). Zhou et al. (2015) hypothesized that the increased expression of NCF2 was associated with muscle remodeling and hypertrophy in the high FE commercial broiler. Thus, the elevation of NCF2 could have a positive effect on muscle development in the high FE phenotype.

**FIGURE 2:**
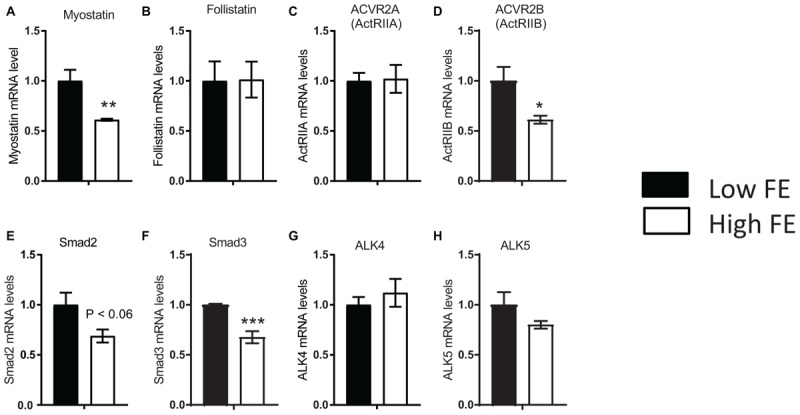
Differentially expressed genes that are involved in myostatin signaling in breast muscle from Pedigree Male (PedM) broilers exhibiting high or low feed efficiency (FE) phenotype. Relative expression of mRNA is shown for myostatin **(A)**, follistatin **(B)**, activin receptor types IIA and IIB (ActIIA and Act IIB) **(C,D)**, Mothers against decapentaplegic homolog 2 and 3 (Smad2 and Smad3) **(E,F)**, and activin receptor-like kinase 4 and 5 (ALK 4 and ALK 5) **(G,H)**. Bars represent the mean + SE (*n* = 5). Mean values were different at ^∗^*P* ≤ 0.05, ^∗∗^*P* ≤ 0.01, or ^∗∗∗^ *P* ≤ 0.001.

**FIGURE 3:**
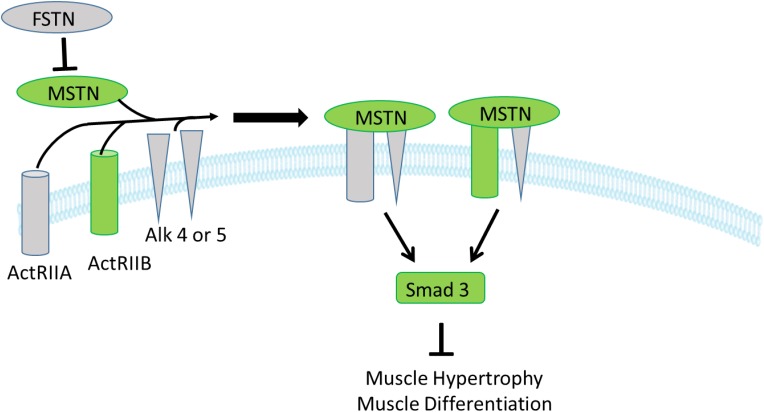
Myostatin signaling in muscle cells. Myostatin initially binds to one of the two activin type II receptors, and then binds to one or both of the activin-like kinase type I receptors 4 and 5 (ALK 4,5). Binding with myostatin activates the ALK4 and ALK 5 type I receptors resulting in phosphorylation of the transcription factors Smad 2,3 that leads to inhibition of muscle growth and differentiation. Gene expression of the molecules shown in green were downregulated in the high FE phenotype (upregulated in the low FE phenotype) whereas molecules in gray were not differentially expressed between the groups. The figure is adapted from Lee and Glass (2011).

In the MSTN signaling pathway, MSTN binds to its receptors, ActIIA or ActIIB that activate ALK4 and ALK5 that in turn phosphorylate Smad 2 and 3 that translocate to the nucleus to initiate changes in transcription of downstream genes; FSTN functions to inhibit and limit MSTN activity (Han and Mitch, 2011; Lee and Glass, 2011). In the present study, expression of the inhibitory gene MSTN (a member of the TGF-β family), one of its receptors (ActRIIB), and two transcription factors of MSTN signaling, SMAD2 and SMAD3, were upregulated in the low FE compared to high FE breast muscle but there were no differences in FSTN, the ActRIIA, ALK4 and ALK5 expression (Figure 2). Since we only examined mRNA expression, we can only assess expression of transcripts and not protein expression or post-translations modifications such as phosphorylation in the MSTN signaling pathway as well as other genes and pathways outlined below. In a microarray study (Kong et al., 2011; Bottje et al., 2012), data analyzed by IPA software predicted that several target molecules and mechanisms may contribute to differences in muscle growth, development, and differentiation between the high and low FE phenotype. Myostatin was downregulated in the high FE phenotype (Bottje et al., 2014). Myostatin is a member of the TGF-β family of molecules and is a strong negative regulator of skeletal muscle growth (McPherron et al., 1997), and differentiation and proliferation of turkey satellite cells (McFarland et al., 2006). The expression of the MSTN antagonist, FSTN, was not different between the two phenotypes (Figure 2B). Studies investigating the relationship between MSTN and growth performance in broilers show that MSTN is a polymorphic gene in which different alleles of the gene can affect performance (Gu et al., 2004; Ye et al., 2007; Bhattacharya and Chatterjee, 2013). Thus, differences in FE in the PedM broiler line in this study could be due in part to different haplotypes of the MSTN gene. Figure 3 (adapted from Lee and Glass, 2011), summarizes the initial steps in the MSTN signaling pathway in the present study that would potentially exert a negative effect on muscle hypertrophy and differentiation in the low FE phenotype.

### Energy Sensing

**FIGURE 4:**
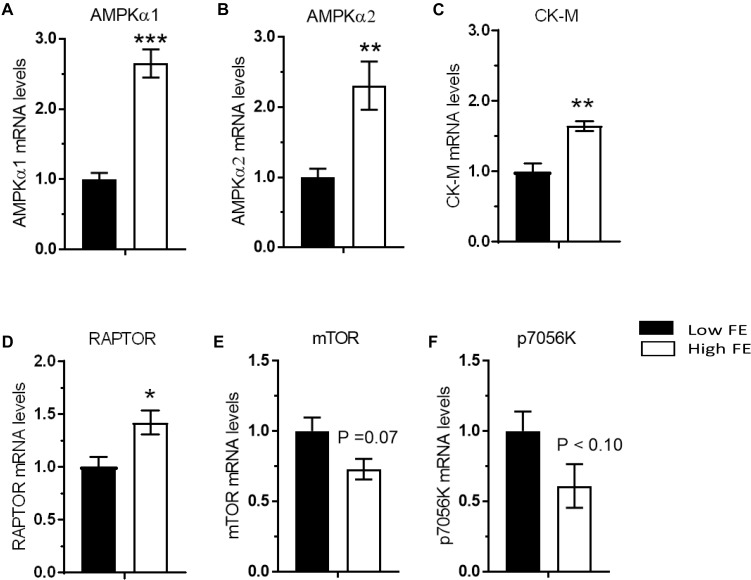
Differentially expressed genes that are involved inmuscle metabolism (energy sensing/storage), and protein synthesis via nutrient sensing in breast muscle from Pedigree Male (PedM) broilers exhibiting high or low feed efficiency (FE) phenotype. Relative expression of mRNA is shown for AMP-activated protein kinase alpha subunits 1 and 2 (AMPKα1) **(A)** and AMPKα2 **(B)**, creatine kinase muscle isoform (CKM) **(C)**, regulatory-associated protein of mTOR (RAPTOR) **(D)**, mechanistic target of rapamycin (mTOR) **(E)**, and 70 kDa ribosomal protein S6 kinase (P70S6K) **(F)**.

Activation of 5′ AMP activated kinase (AMPK) occurs via phosphorylation and allosteric modification by 5′-AMP in response to an increase in the cellular AMP:ATP ratio (Carling et al., 1987). This produces a cellular response in which energy-consuming reactions are inhibited and energy-producing reactions are enhanced. Both isoforms of the catalytic subunit of 5′ AMP activated protein kinase (AMPKα1 and AMPKα2) were upregulated in the high FE phenotype (Figure 4A,B) which verifies the previous results (Bottje et al., 2012). AMPKa was also predicted to be activated in the high FE PedM phenotype in a proteomics study conducted on the same muscle samples (Kong et al., 2016) and both AMPKa 1 and AMPKa 2 were predicted to be activated in an RNAseq study (unpublished observations). AMPK expression increases in response to low energy levels that increases ATP production by stimulating mitochondrial biogenesis and oxidative phosphorylation, glycolysis and lipolysis, and inhibiting fatty acid synthesis, protein synthesis, and gluconeogenesis that consume ATP (Zhou et al., 2001; Hardie et al., 2003; Carling, 2004).

Interestingly, creatine kinase (muscle isoform, CK-M) gene expression was elevated in the high FE phenotype (Figure 4C) and concurs with CK-M expression in a transcriptomic study (Bottje et al., 2017a). Although the brain isoform of CK (CK-B) was elevated in the high FE PedM phenotype, CK-M protein was higher in the low FE phenotype (Kong et al., 2016; Bottje et al., 2017b). The reason for this discrepancy is not apparent but gene and protein expression often do not go hand in hand. Nonetheless, increased expression of several proteins suggests that high FE phenotype PedM broiler breast muscle may have enhanced capabilities for mitochondrial oxidative phosphorylation as well as the ability to shuttle creatine and phosphorylated creatine in and out of mitochondria (Bottje et al., 2017b).

### Protein Synthesis

When active, the mTORC1 complex functions to promote cell growth by increasing protein synthesis and lipid metabolism, inhibiting autophagy, and also modulates the transcription of several genes (Laplante and Sabatini, 2013). In a cDNA microarray study, mTORC1 expression was higher in the high FE phenotype (Bottje et al., 2014). Two major components of mTORC1 complex are mTOR and regulatory associated protein of mTOR (RAPTOR) (Kim et al., 2002). In the present study, RAPTOR was upregulated (*P* < 0.05) in the high FE birds (Figure 4D) and the mTOR was moderately upregulated (*P* < 0.07) in the low FE birds (Figure 4E,F). The increase in RAPTOR gene expression could have a positive effect on protein synthesis (Kim et al., 2002). In humans, mTOR expression is dysregulated during disease states such as cancer, diabetes, and heart disease (Dowling et al., 2010) that all have a commonality of increased mitochondrial ROS production. Thus, elevated mitochondrial ROS reported in low FE PedM broilers (Bottje and Carstens, 2009) might explain the upregulation of mTOR and p70S6k seen in the low FE phenotype. This observation may also hold true for PRKAR1A and GLUT-8, two other members of the insulin signaling pathway that were upregulated in the low FE phenotype (see below). The moderate decrease in mTOR expression in this study contrasts with a significant elevation in mTOR expression observed in high FE PedM broilers (Piekarski-Welsher et al., 2018). We do not have an explanation for this discrepancy between these studies at this time. As indicated previously, we did not assess protein expression in the present study and so do not know if there were differences between FE phentoypes in the phosphorylated (active) form of mTOR in the present study.

Key downstream targets of mTORC1 involved in enhancing protein synthesis are p70S6k and eukaryotic translation initiation factor 4E (EIF4E). The expression of p70S6k, which is activated by insulin and refeeding (Bigot et al., 2003), was higher in the low FE birds (*P* < 0.08) (Figure 4F). Examination of unreported RNAseq transcriptomic data conducted on the same set of muscle tissue (from Bottje et al., 2017a) revealed a significant skew (binomial *P*-value = 0.0002) favoring greater abundance of eukaryotic initiation, elongation, and translation genes with numerically higher expression in the high FE compared to the low FE PedM phenotype (Table 2). This finding, combined with enrichment of ribosomal machinery (Bottje et al., 2017c), indicates that protein synthesis infrastructure is enhanced in the high FE phenotype.

### Insulin Signaling

**FIGURE 5:**
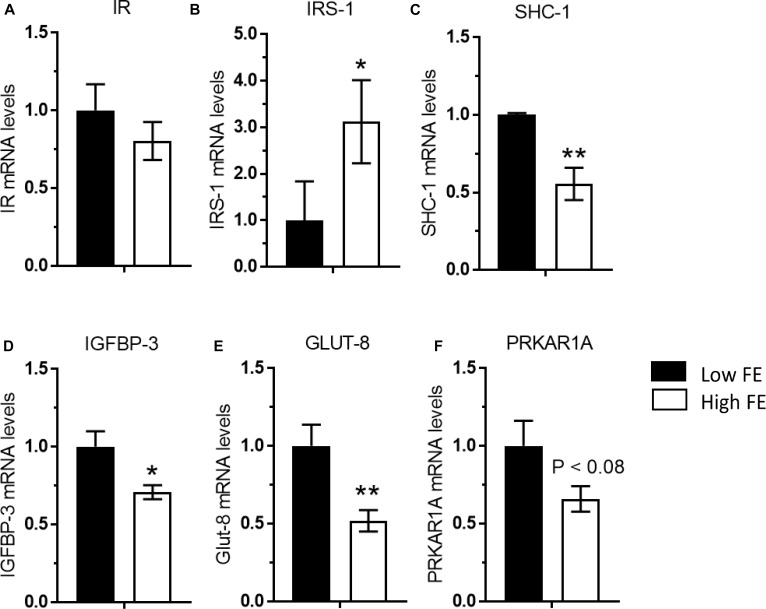
Gene expression associated with the insulin signaling pathway in breast muscle from Pedigree Male (PedM) broilers exhibiting high or low feed efficiency (FE) phenotype. Relative expression of mRNA is shown for; **(A)** insulin receptor (IR), **(B)** insulin receptor substrate 1 (IRS-1), **(C)** SHC-transforming protein 1 (SHC-1), **(D)** insulin-like growth factor-binding protein 3 (IGFBP-3), **(E)** glucose transporter 8 (GLUT-8), and **(F)** cAMP-dependent protein kinase type I-alpha regulatory subunit (PRKAR1A). Mean values were different at ^∗^*P* ≤ 0.05 or ^∗∗^*P* ≤ 0.01.

The results of targeted mRNA expression of genes associated with insulin signaling are presented in Figure 5. Insulin signaling and its pleiotropic effects on gene expression and muscle development in chickens is not as thoroughly understood and differs in certain ways when compared to mammals (Dupont et al., 2015). Unlike mammals, in chicken muscle only SHC-1 (not IRS-1) is activated by changes in nutritional status, suggesting that chickens have a tissue-specific regulation of insulin signaling that is yet to be fully understood (Dupont et al., 1998a,b). Although there was no difference in insulin receptor gene expression (Figure 5A), insulin-like substrate 1 (IRS-1) was upregulated in the high FE birds (Figure 5B), which concurs with the predicted activation of IRS-1 and insulin like growth factor receptor 1 in Kong et al. (2016). Additionally, insulin signaling requires insulin receptor endocytosis and is particularly dependent on CAV-1 (Cohen et al., 2003). CAV-1 protein expression was ninefold higher in the high FE compared to low FE breast muscle (Kong et al., 2016) and could be instrumental in facilitating insulin signaling in the high FE PedM broiler.

It has been suggested that p70S6k is involved in a negative feedback that inhibits IRS-1 activation by phosphorylating its serine residues (Duchene et al., 2008). Since p70S6k was marginally upregulated (*P* < 0.08) in the Low FE phenotype in the current study, it may be inhibiting IRS-1 activity, and thus increased SHC-1 expression (Figure 5C) would help maintain the insulin signaling pathway in low FE. Both IRS-1 and SHC-1 are activated by tyrosine phosphorylation activity mediated by phophoinositide-3 kinase (PI3K) when insulin binds to the insulin receptor. We did report an elevation in PI3K expression in the high FE phenotype in a cDNA microarray study (Bottje et al., 2012) that could be hypothesized to activate both IRS1 and SHC-1.

Insulin degrading enzyme plays a role in insulin signaling and insulin activity (Leissring et al., 2010; Fawcett, 2014). In addition, a mitochondrial form of IDE is capable of cleaving mitochondrial leader signals of nuclear DNA-encoded mitochondrial proteins (Leissring et al., 2004), thus making the enzyme important in mitochondrial function. Although there was no difference in mRNA expression of IDE in the present study (data not shown), IDE protein expression was upregulated in the high PedM broiler phenotype (Kong et al., 2016).

The upregulation of anti-proliferative IGFBP-3 in the low FE phenotype (Figure 5D) concurs with Zhou et al. (2015). IGFBP3 may modulate the interaction of insulin like growth factor (IGF) in the extracellular matrix (Stewart and Rotwein, 1996). Spangenburg et al. (2003) reported that IGFBP3 (both gene and protein) was present in rat soleus muscle (Type 1 muscle fiber) but not in Type I and II muscle fibers in gastrocnemius muscle. IGFBP3 was reported to play a role in differentiation in human myoblasts (Foulstone et al., 2003).

Along with stimulating mitochondrial biogenesis, AMPK increases expression of glucose transporter 4 (GLUT 4), glucose transport and fatty acid oxidation in skeletal muscle in mammals (e.g., Balon and Jasman, 2001; Smith et al., 2005). Thus, it is somewhat surprising that with the up-regulation of AMPKα1 and AMPKα2 (Figure 4A,B), that both glucose transporter (GLUT 8) and PRKAR1A (Protein Kinase cAMP-dependent type 1 regulatory subunit alpha) were down regulated in the high FE phenotype (Figure 5E,F). High circulating levels of glucose down-regulate GLUT4 expression in many tissues ranging from muscle to white blood cells in mammals (e.g., Klip et al., 1994; Li et al., 2004; Kiomen-Korgun et al., 2009). Thus, the high FE phenotype may have higher circulating levels of glucose compared to the low FE phenotype. PRKAR1A is the main component of PKA that regulates most serine-threonine kinase activity in response to increases in c-AMP including lipolysis (see review Bossis and Stratakis, 2004) which is enhanced by AMPK. Thus, additional research is warranted to understand the link between increased AMPK expression and down-regulation of in the high FE phenotype.

### Summary

**FIGURE 6:**
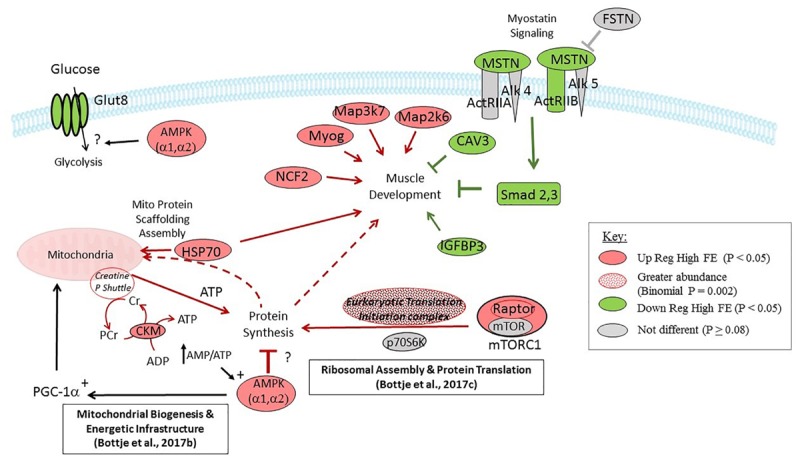
Diagrammatic representation of targeted gene expression analysis conducted in the present study. Genes shown in red and green were up- and down-regulated (*P* < 0.05), respectively, in breast muscle in the high compared to the low feed efficiency pedigree male broiler phenotype. Genes shown in gray were not differentially expressed (*P* > 0.08). The eukaryotic translation and initiation complex exhibited greater abundance in the high FE PedM phenotype (Table 2, bionomial *P* = 0.0002). Gene expression that would enhance muscle development in the high FE phenotype include the upregulation of Myog, Map3K7, Map2K6, and NCF2 and down regulation of CAV3 and Smad 2,3. Components that would potentially enhance protein synthesis include Raptor and eukaryotic translation and initiation complex expression (see Table 2) and components supporting ribosome assembly and mRNA translation (see Bottje et al., 2017c). Increased CK-M combined with energetic infrastructure and proteins associated with creatine kinase shuttle expression (Bottje et al., 2017b) could provide ATP needed for enhanced protein synthesis in high FE breast muscle. Aspects that are not clear in this study are the potential negative effect of AMPK on protein synthesis and downregulation of the Glut 8 receptor in the high FE phenotype.

A mechanistic picture of gene expression data in this study is depicted in Figure 6. Muscle development in the high FE PedM phenotype would be fostered by a combination of; (a) down regulation of expression of MSTN, Smad2 and Smad3 in the myostatin signaling pathway combined with down regulation of CAV3 and IGFBP3, and (b) up regulation of HSP70, NCF2, MYOG, Map2k7, and Map2k6. Synthesis of breast muscle proteins could be enhanced in the high FE phenotype by increased expression of RAPTOR in the mTORC1 complex and increased abundance of genes associated with the eukaryotic translation and initiation complex. The expression of mTORC1 was also reported to be upregulated in the high FE phenotype in a previous study (Bottje et al., 2014). Potential negative effects on protein synthesis activities by moderate down-regulation of mTOR and p70S6K could be offset by the enhanced ribosomal assembly and protein translation infrastructure reported previously (Bottje et al., 2017c). Protein scaffolding and assembly of nuclear DNA encoded proteins that are imported into the mitochondria would be enhanced by increased HSP70 expression in the high FE phenotype. Although protein synthesis might be inhibited by increased expression of AMPK (which responds to energy demand signals such as an increase in the AMP:ATP ratio) in the high FE phenotype, we hypothesize that AMPK-mediated stimulation of peroxisome proliferator activated receptor gamma coactivator 1 alpha (PGC-1α), along with enhanced CKM and energetic infrastructure in the high FE phenotype (Bottje et al., 2017b), would provide sufficient ATP to support protein synthesis in the high FE phenotype. Expression of AMPK is well-known to be part of an energy sensing system that responds to increased energy demand signals such as an increase in the AMP:ATP ratio that activates PGC-1α in muscle cells (e.g., Lira et al., 2010). In turn, PGC-1α has been given the title of master regulator of mitochondrial biogenesis by Nisoli et al. (2003) and therefore instrumental in increasing energy production through increased mitochondrial oxidative phosphorylation to meet increased energy demand in the cell.

There are at least two inconsistencies in this study that need to be pointed out. As indicated previously, AMPK expression is elevated in response to energy demand that would increase energy generating pathways (such as lipolysis and glycolysis) and mitochondrial oxidative phosphorylation (through mitochondrial biogenesis as mentioned previously). Thus, the down regulation of GLUT 8 (*P* < 0.05) and PRKAR1A (*P* = 0.08) that are important genes in glycolysis and lipolysis, respectively, is puzzling. This apparent discrepancy could be due in part because this study focused on gene expression and did not entail targeted protein expression. For example, insulin signaling was predicted to be activated in the high FE phenotype in a proteomics study (Kong et al., 2016) whereas gene expression analysis in this study did not reveal a clear indication of enhanced insulin signaling in the present study.

## Author Contributions

KL, BK, SD, and WB designed and/or conducted the studies. This manuscript is part of KL Ph.D. Dissertation. The analysis of data was carried out by the same authors with A-PW. The paper was written through contributions and critical review by all authors.

## Conflict of Interest Statement

AP-W is employed by Adisseo USA, Alpharetta, GA, United States. The remaining authors declare that the research was conducted in the absence of any commercial or financial relationships that could be construed as a potential conflict of interest.
